# Low level of Vitamin C and dysregulation of Vitamin C transporter might be involved in the severity of COVID-19 Infection

**DOI:** 10.14336/AD.2020.0918

**Published:** 2021-02-01

**Authors:** Taylor Patterson, Carlos M Isales, Sadanand Fulzele

**Affiliations:** ^1^Department of Medicine, Augusta University, Augusta, GA 30912, USA.; ^2^Center for Healthy Aging, Augusta University, Augusta, GA 30912, USA; ^3^Department of Cell biology and anatomy, Augusta University, Augusta, GA 30912, USA

**Keywords:** Vitamin C, Coronavirus, Aging, Immune response

## Abstract

The Severe Acute Respiratory Syndrome Coronavirus 2 (SARS-CoV-2) has been spreading around the world at an exponential pace, leading to millions of individuals developing the associated disease called COVID-19. Due to the novel nature and the lack of immunity within humans, there has been a collective global effort to find effective treatments against the virus. This has led the scientific community to repurpose Food and Drug Administration (FDA) approved drugs with known safety profiles. Of the many possible drugs, vitamin C has been on the shortlist of possible interventions due to its beneficial role as an immune booster and inherent antioxidant properties. Within this manuscript, a detailed discussion regarding the intracellular function and inherent properties of vitamin C is conducted. It also provides a comprehensive review of published research pertaining to the differences in expression of the vitamin C transporter under several pathophysiologic conditions. Finally, we review recently published research investigating the efficacy of vitamin C administration in treating viral infection and life-threatening conditions. Overall, this manuscript aims to present existing information regarding the extent to which vitamin C can be an effective treatment for COVID-19 and possible explanations as to why it may work in some individuals but not in others.

The ongoing global Coronavirus pandemic, also known as COVID-19, has had a significant impact on world health. Within a time frame of 7 months, positive cases of COVID-19 have reached 7.2 million and more than 400 thousand deaths (at the time of writing) recorded worldwide according to the online database provided by Johns Hopkins University [1]. The limited knowledge and unique nature of the virus have left clinicians scrambling to find effective treatments for severely ill patients, as well as hindering the development of preventive measures to slow the exponential spread of the virus. This has led scientists and physicians to repurpose drugs approved by the Food and Drug Administration (FDA) with known safety profiles.

Intravenous high dose vitamin C therapy is among the shortlist of potential drug regimens being tested for efficacy in the treatment of COVID-19 [2-4]. Several researchers and clinicians have hypothesized that the use of ascorbic acid could reduce SARS-CoV-2 infection via the supplements ability to boost immune response along with diminishing the severity of the viral-mediated inflammatory response. A number of studies support the finding that a high dose of the vitamin helps boost the immune system [5, 6]. Still, there are several important variables for which there is little information, including the impact of aging, vitamin C transporter system, gender, and race. Therefore, this paper aim is to summarize previously published data pertaining to the effects of vitamin C administration/supplementation on the immune response, disease prevention, and progression. We also discuss the role of age, vitamin C transporter systems, gender, and race, and their impact on the effectiveness of vitamin C in diseased conditions. COVID-19 infection disproportionally affects older individuals, males, and specific populations, including African American and Hispanic groups [7]. Therefore, it is important to consider and thoroughly investigate the factors mentioned above before arriving at any conclusion on the effectiveness of vitamin C treatment.

## General Background on Vitamin C

Vitamin C, also known as ascorbic acid, is one of the essential vitamins needed for mammalian species to survive and thrive. Through evolution, the *homo sapiens* species has lost the ability to synthesize vitamin C due to the inactivation of the gluconolactone oxidase gene [8]. However, fruits and vegetables such as strawberries, oranges, and broccoli are rich in vitamin C and readily available for human consumption. Due to its water-soluble nature, most vitamin C is absorbed across the intestinal lumen and transported across cellular membranes via sodium-dependent vitamin c transporter 1 and 2 (SVCT 1 and 2) depending upon tissue type. There is an alternative way that the vitamin can gain access to the intracellular space, that being via the Glucose Transporters (GLUTs). However, the vitamin must be in the oxidized form (dehydroascorbic acid) for this to occur. Vitamin C's unique chemical properties, such as being an electron donor/reducing agent, allow it to have antioxidant properties and act as a coenzyme for more than fifteen mammalian enzymatic reactions [9]. These enzymes include monooxygenases (Dopamine β-Hydroxylase critical for norepinephrine synthesis), dioxygenases (Prolyl-Hydroxylase and Lysyl Hydroxylase), and amine oxidase [10]. Perhaps one of the most well-known actions that vitamin C participates in involves hydroxylation of proline and lysine residues during the synthesis of collagen [9].

Low levels of vitamin C can cause a myriad of problems, with prolonged deficiencies leading to scurvy, a disease often associated with sailors in the 1800s (due to lack of fresh fruits and vegetables) while at sea. Symptoms such as bleeding gums, abnormal wound healing, and fever are commonly associated with the disease and can be attributed to the inability of certain enzymes to function properly, especially those involved in collagen synthesis. Furthermore, it has been noted from previous studies that patients suffering from various pathophysiological conditions such as diabetes, COPD, chronic hypertension and viral induced sepsis, have decreased levels of serum and plasma vitamin C [11-14]. This has led to studies on the use of intravenous administration of vitamin C for the treatment of patients suffering from severe and chronic diseases as well as viral infections such as COVID-19.

## Vitamin C Levels and Immune System

It has been noted from previous studies that resting neutrophils contain high intracellular levels of vitamin C, around 1-2 mM, or about 10-100 fold higher than average plasma levels [15]. This intracellular concentration only increases when neutrophils are activated and begin to undergo oxidative burst, with levels reaching 10-20 mM following stimulation [15, 16]. Due to this phenomenon and vitamin C's antioxidant properties, it has been hypothesized that vitamin C plays a vital role in neutrophil function and thus essential for proper immune system response. In order to investigate this hypothesis, Bozonet et al. (2015) [6] conducted a cohort study including fourteen men (aged 18-30) for four weeks in which they maintained a regimen eliminating juice and high vitamin C foods, such as citrus and kiwifruit, from their diet to achieve suboptimal plasma vitamin C levels (<50 μmol/L). Following the initial four weeks "lead-in" period, baseline data was obtained via a blood sample. Plasma vitamin C and neutrophilic superoxide levels were measured, as well as neutrophil chemotaxis assay performed. The men were then given two gold kiwifruit (~256 mg Vit C) a day for four weeks. Blood samples were then drawn again following the four weeks [6]. They reported that the mean levels of serum vitamin C and neutrophilic vitamin C substantially increased from baseline to post-intervention (26±3-72±2, 21±1-26±2 mM; respectively). Mean neutrophil chemotaxis showed an increase of 20% (*p* = 0.041) as well as an increase in superoxide production of 23% (*p* = 0.031) post-intervention. The end results showed that adequate vitamin C levels are imperative for proper neutrophil function and normal immune system function [6]. It is known that in patients with severe COVID-19 infection, hyperactivity within the immune system is rampant. However, there are indications that providing excessive vitamin C in those with deficient levels will allow for proper immune function, thus limiting and decreasing the severity of the infection.

## Regulation of Vitamin C Transporter with Aging and Pathophysiological Conditions

Vitamin C is highly water-soluble; it cannot directly diffuse across the hydrophobic lipid bilayer of the plasma membrane to gain access into cells. A specific transport system, sodium-dependent vitamin C transporters (SVCT), exists in the plasma membrane to mediate the entry process. There are two subtypes of sodium-dependent vitamin C transporter, SVCT1, and SVCT2 [17-20]. The expression of vitamin C transporters is cell and tissue-specific. Sodium-dependent vitamin C transporter 1 is expressed in a number of different tissue and cell types, including liver, kidneys, and intestinal epithelial cells, whereas SVCT2 transporter is expressed in various tissues such as liver, brain, heart, chondrocytes, and osteoblast [17-19].

**Table 1:** Selected studies showing dysregulation of vitamin C transporter (SVCT1 and SVCT2) in different pathological condition or stress.

Author	Study Type	Pathophysiological condition	Vitamin C regulation
Macias et al. 2010	Human study on liver biopsies	hepatocellular cholestasis, primary biliary cirrhosis, haemochromatosis and non-alcoholic steatohepatitis	-Increase SVCT 1 and SVCT 2 expression
Blackburn AJ et al. 2014	Human Osteoarthritis	Osteoarthritis	SVCT2 downregulated in OA grade 3 tissue compared to OA grade 1
Macias et al. 2010	Animal study	Obstructive Cholestasis	-Increase SVCT 2 expression -Decrease SVCT 1 expression
Miao et al. 2019	Animal cell culture study	Melanocyte exposure to excess vitamin	-Increase SVCT 2 expression -No change SVCT 1 expression
Kang et al. 2007	Human cell culture study	UVB damaged keratinocytes	-No change in SVCT 1 and 2 expression
Subramanian et al. 2016	Human cell culture and animal study	Chronic alcohol exposure	Human cells: decrease SVCT 2 expression Animal cells: decrease SVCT2 expression
Wu et al. 2007	Animal study	Streptozotocin (STZ)-induced diabetes	-Increase SVCT 2 expression in adrenals -Decrease SVCT 1 expression in kidney
Michels et al. 2003	Animal study	Aged rats	-Decrease in hepatic SVCT 1 expression -No change in hepatic SVCT 2 expression in aged rats
Bayram et al. 2013	Animal study	Fast aging phenotypic mice	-Increase in hepatic SVCT 1 expression -No change in hepatic SVCT 2 expression
Sangani et al. 2013	Animal Model	Diabetic	Decrease SVCT2 in bone and bone marrow
Sangani et al. 2015	Cell culture	Apoptosis and Autophagy	SVCT2 regulates Apoptosis and Autophagy

Several studies reported that aging, oxidative stress, and inflammatory factors cause changes in the expression of both SVCT 1 and 2 [17, 21]. Only a few human studies reported the regulation of vitamin C transporter in pathophysiological conditions. In 2014, our lab demonstrated that the SVCT2 transporter expression is significantly down-regulated in osteoarthritic tissue compared to healthy tissue [22]. However, expression of the vitamin C transporter is not altered in all pathological conditions. Larsson et al. (2015) reported no differences in SVCT 1 and 2 expressions in alveoli tissue, macrophages, lymphocytes, and neutrophils when comparing asthmatics to healthy subjects [23]. But, there are several *in vitro* and animal studies demonstrating drastically altered expression of vitamin C transporter (SVCT1 and SVCT2) in the presence of oxidative stress, inflammatory factors, and various disease conditions (See Table 1) [22, 24-29]. Further research into the regulation of the SVCT expression under various pathophysiological conditions is warranted in order to better understand whether these alterations are normal fluctuations, or abnormal due to underlying pathology caused by the condition.

With the advancement in genome sequencing technology, some studies reported polymorphisms in transporter regions that have shown correlations with significant declines in plasma vitamin C levels despite high dietary vitamin C intake [30-32]. Researchers found that the SNP (rs33972313) is associated with a 50% decline in the rate of ascorbate accumulation in cells. Interestingly, this polymorphism was found to be present at a higher frequency in African, African American, and Yoruba African populations [33-35]. We speculate that dysregulation of SVCT transport and genetic polymorphism might be important contributing factors in many age-related pathophysiological conditions and increase COVID-19 infection and severity in certain sections of the populations.

## Vitamin C Level Disparity in Gender and Race

In the past decade, several research groups conducted studies investigating whether serum and plasma vitamin C levels differed between gender and race. The data collected from those studies were surprising. In almost all studies, there was a statistically significant difference in average plasma vitamin C concentration between males and females, with male plasma vitamin C levels much lower than in females despite a diet consisting of higher levels of vitamin C [36-39]. Data also showed disparities in vitamin C levels among different races/ethnicities. Non-Hispanic African Americans had a much larger chance of having vitamin C deficiency compared to Caucasian Americans; however unlike in the gender data, average dietary vitamin C intake was much lower in the non-Hispanic African American participant group [37, 38]. It should be noted that dietary recall, an often-misreported data collection method, was used to determine the dietary vitamin C levels of the respective groups and thus could have affected the final outcomes from these respective trials.

**Table 2:** Risk factors for developing severe covid-19 infections and relationship to serum vitamin C levels.

Gender	Relative Serum Vit C Concentration	Relative Chance of Severe Covid-19
Male	+ + [37, 38]	+ + + [7, 40]
Female	+ + + [37, 38]	+ + [7, 40]
Race		
Caucasian	+ + + [37, 38]	+ + [7, 41]
African American	+ + [37, 38]	+ + + [7, 41]
Underlying Condition		
Diabetes	+ + [13]	+ + + [7]
Hypertension	+ + [42, 47]	+ + + [7]
COPD	+ + [36, 45]	+ + + [7, 44]
No Complication	+ + + [37, 38]	+ [7]
Age		
0-44	+++ [14, 42]	+ [7]
45-64	++ [14, 42]	++ [7, 44]
≥65	+ [14, 42]	+++ [7, 44]
“+” = arbitrary unit		

It well know that COVID-19 infection rate and severity have been disproportionately higher in males, indicated by the drastic increase of mortality rates in males compared to females in hard hit countries such as Spain and Italy [7, 40]. Furthermore, non-Hispanic African Americans also exhibited a higher prevalence rate and severity compared to Caucasian Americans [7, 41]. Johns Hopkins reported that of the 131 predominately black counties in America, the infection rate of COVID-19 was 3-fold higher and the death rate was 6-fold higher than in predominately white communities [41]. There is a possibility that vitamin C levels might be one of the factors accounting for the higher prevalence and severity of COVID-19 infection in male and non-Hispanic African Americans. Table 2 shows the correlation between vitamin C levels and the risk of COVID-19 infection and mortality. However, ultimately determining whether this correlation is directly related warrants further research.

## Vitamin C levels in the Aged and with Underlying Conditions

Vitamin C levels have been shown to decrease in the elderly population as well as those exhibiting chronic underlying conditions (e.g. diabetes, hypertension) [42]. Fletcher et al. (2003) conducted an interesting study to correlate the association between vitamin C levels and mortality in older persons [14]. They reported a strong inverse correlation with blood ascorbic acid levels and all-cause mortality, including cardiovascular disease. Those patients in the lowest quantile for blood ascorbic acid (<17 μmol/L) showed the highest mortality rate with a hazard ratio of 1. Those in the highest quantile (>66 μmol/L) showed the lowest mortality rate indicated by a hazard ratio of half that (.54). Fletcher et al. (2003) concluded that serum vitamin C levels are a strong predictor of mortality in the aged population [14]. From this study, it seems that vitamin C levels are positively correlated with longevity.

Several studies demonstrated a decline in vitamin C levels in most of the underlying conditions [13]. Hypertension and diabetic conditions are the risk factors to increase COVID-19 infection and mortality [7]. Wilson et al. (2017) investigated vitamin C levels in healthy individuals and type-2 diabetic patients [13]. They reported significantly lower plasma vitamin C levels in T2D patients compared to healthy individuals (41.2 µmol/L vs. 57.4 µmol/L, p < 0.05) [13]. Low serum vitamin C levels lead to disruption in the normal distribution of ascorbic acid to a certain tissue in the body, thus leading to many of the frequently seen diabetic complications such as hyperlipidemia, neuropathy, and hyperglycemia [13, 43].

The prevalence and severity of COVID-19 infection are drastically higher within the elderly population group and the underlying condition, as mention above [7, 44]. However, no study has linked decreasing levels of vitamin C with increased susceptibility and severity of COVID-19 infection. A study looking at this associated would help us to better understand the increased virulence of COVID-19 in these populations and whether vitamin C therapy will have a substantial therapeutic benefit in treating the disease.

## Vitamin C Supplementation for Underlying Conditions

Antioxidants supplementation is essential for reducing inflammation by decreasing proinflammatory cytokine production. In COPD patients, chronic elevated oxidative stress and inflammatory cytokines are major contributors to the pathogenesis and decreased respiratory function seen in these patients [45]. Previously, MacNee et al. (2000) [46] conducted a single-blinded randomized control trial aimed to evaluate the efficacy of vitamin C and/or N-acetylcysteine (NAC) supplementation in increasing antioxidant status in COPD patients [45]. A total of 79 patients who had previously been diagnosed with COPD were enrolled in this trial and divided into four groups. The first group received IV NAC (600 mg/day), the second group received IV vitamin C (500 mg/day), the third group received IV NAC (600 mg/day) + IV vitamin C (500 mg/day), and the fourth group received a placebo solution. The patient's glutathione levels, a reliable indicator of antioxidant status, was measured at baseline, 3 months, and 6 months post-treatment [45]. Results showed that group 2 had the greatest increase in glutathione levels compared to the control group, with an increase of 516% compared to 56% increase (P=0.005) following 6 months of treatment. They concluded that NAC supplementation alone showed a dramatic improvement in the nutritional status of COPD patients, while Vitamin C supplementation alone showed a remarkable increase in antioxidant status [45].

As mentioned above, hypertension is associated with decreased serum vitamin C levels, so it is logical to propose supplementation of vitamin C to treat hypertension. Several clinical studies have been published using vitamin C with mixed outcomes [47, 48]. A study published in 2012 performed a Meta-Analysis of Randomized Controlled Trials investigating the effects of vitamin C supplementation on blood pressure from 1966 to 2011 (included 29 clinical trials) and showed a positive outcome of vitamin C treatment in reducing SBP and DBP. However, after analyzing the data from the trials, it was noted that the average change in blood pressure was small (> 5 mm hg) [48]. Ghosh et al. (1994) performed aged and sex-match randomized double-blind study to treat hypertensive subjects with vitamin C supplementation in aged patients [47]. The participants received either oral Vitamin C 250 mg once or twice daily or a placebo for 6 weeks, followed by analysis on plasma vitamin C and lipid peroxidase. They reported a significant fall of systolic and diastolic blood pressure in the treatment group, but surprisingly, no statistical difference in blood pressure between treatment and placebo groups [47]. Ghosh et al. (1994) conclude that vitamin C treatment showed marked antioxidant action but need to investigate thoroughly for any hypotensive action [47]. Similarly, the mixed benefits of vitamin C supplementation have been reported in diabetic clinical trials [49-51].

**Table 3:** Selected studies pertaining to the efficacy behind Vitamin C supplementation for patients with diseases and/or viral infections.

Author	Viral infection	Age	Dose	Outcome
Zabet et al. 2016 Type: RCT	Sepsis	18-65 years old	-Vitamin C (25mg/kg) every 6 hours	-28 day mortality was significantly lower (14.28% vs. 64.28%, respectively; *P* = 0.009)
Fowler et al. 2019 -Type: double blinded, RCT	Patients with Sepsis or ARDS for less than 24 hours	Mean age of 54.8 years old	-IV infusion of vitamin C (50 mg/kg in dextrose 5% in water, n=84) every 6 hours	-No significant difference in mean modified SOFA score, C-reactive protein or thrombomodulin levels -HOWEVER, strong indication of lower all-cause mortality in vit C group
Sawyer et al. 1986 Type: RCT	Patients with ARDS	N/A	-IV injection of vitamin C (1000 mg) every 6 hours	-Dramatic reduction in mortality in vit. C group compared with control (37% vs. 71% (p<.01))
Fowler et al. 2014 Type: Double Blinded, RCT	Patients in MICU with severe Sepsis	30-70 years old in low dose group 49-92 years old in high dose group 54-68 years old in placebo group	-Low dose group: IV Vitamin C (50 mg/kg/24 h, n=8) -High dose group: IV Vitamin C (200 mg/kg/24 h, n=8) -Placebo group: IV (5% dextrose/water, n=8)	-SOFA score -Hi: 10.4±4.4 -Lo: 10.1±2.0 -Placebo: 13.3±2.9 -Vit. C groups decreased levels of C-reactive protein and procalcitonin -No significant difference between thrombomodulin levels between groups
Marik et al. 2017 Type: Retrospective clinical study	Patients diagnosed with severe Sepsis of septic shock	Study group mean age was 58.3 years old Control group mean age was 62.2 years old	-Patients treated with triple therapy of hydrocortisone, HDIVC, Thiamine	-Mortality rate -Treatment: 8.5% (4 of 47) - Control: 40.4% (19 of 47) *(P < .001) -72 hr ΔSOFA -Treatment: 4.8 ± 2.4 -Control: 0.9 ± 2.7 *(P<.001)
Fujii et al. 2020 Type: RCT	Patients in ICUs suffering from Sepsis	Mean age 61.7 years old	-Intervention group: IV vitamin C (1.5 g every 6 hours), hydrocortisone (50 mg every 6 hours), thiamine (200 mg every 12 hours) -Control group: IV hydrocortisone (50 mg every 6 hours)	-No difference in time alive and free of vasopressor administration up to 7 days between intervention group and control group (122.1 hours vs. 124.6 hours; respectively) -ninety-day mortality -28.6% (intervention group) vs. 24.5% (control group)
Hemilä et al. 2013 Type: meta-analysis	Patients suffering from the common cold	N/A	-First arm: 29 trials with vitamin C supplementation (>.2 g/day) -Second arm: 31 trials with regular vitamin C intake (>.2 g/day) -Third arm: 7 trials with therapeutic use of IV or oral vitamin C (>.2 g/day)	-First arm -Risk Ratio of .97 -Second arm -Regular vit C reduced cold duration by 8% in adult population studied -Regular vit C reduced cold duration by 12% in children population studied -Third arm: no consistent effect of therapeutic use of vit C
Fowler et al. 2017 Type: case report	Single patient presenting with enterovirus/rhinovirus induced ARDS	20 years old	-high does Intravenous vitamin C injections (200 mg/kg per day)	-12 hours following initiation of treatment, symptoms dramatically improved -mechanical ventilation was discontinued 7 days post treatment -No long term ARDS sequelae noted

## Vitamin C Supplementation for Sepsis

Clinical studies looking at severe cases of COVID-19 infection that resulted in death have quickly realized that the virus induces a rapid increase in proinflammatory cytokines and chemokines [52]. Without proper intervention, this can quickly compound into viral-induced sepsis causing full-blown cytokine storm for the patient, and ultimately resulting in severe injury or death. Acute respiratory distress syndrome (ARDS) is one of the severe complications of sepsis [53]. Therefore, it has been proposed that therapeutic intervention available for sepsis might be repurposed for the treatment of COVID-19. It has previously been noted that low vitamin C serum levels correlate with worse outcomes in septic patients [54], which gives a strong indication that vitamin C injection/supplementation might be beneficial. Several clinical studies used vitamin C to treat patients suffering from sepsis and acute respiratory distress syndrome (Table 3).

The first clinical study performed by Sawyer et al. (1986) in which 16 patients suffering from acute respiratory distress syndrome (ARDS) were treated with vitamin C (1000 mg IV every 6 h) plus antioxidants (N-acetylcysteine, selenium, and vitamin E) versus 16 ARDS patients who received the standard care at that time. They reported a dramatic reduction in end case mortality in the vitamin C group compared with the control group (37% vs. 71%) [55]. Syed et al. (2014) conducted a phase I clinical trial in which vitamin C injections were given to investigate the efficacy of preventing multiple organ failure in patients suffering from severe sepsis. They reported that the group receiving vitamin C infusions showed far less organ failure compared to the control group [56]. The benefits of vitamin C was also exhibited in a single case review of a patient presenting to the emergency department suffering from enterovirus/rhinovirus induced acute respiratory distress syndrome [57]. After mechanical ventilation failed to stabilize the patient, venovenous extracorporeal membrane oxygenation (ECMO) along with IV administration of vitamin C (200 mg/kg per day) was initiated. Lung gas exchange and lung opacities on x-ray significantly improved following the new course of treatment, with the extubation from ventilation occurring at day 7 post-intervention [57].

The recently published randomized, double-blind, placebo-controlled, multicenter (CITRIS-ALI ) trial that took place between September 2014 to November 2017 to study the effect of vitamin C (intravenous infusion) on organ failure score, inflammatory markers and all-cause mortality in patients with sepsis and ARDS [58]. A group of 167 patients met the diagnostic criteria and were enrolled in the trial with half receiving an intravenous infusion of vitamin C (50 mg/kg in dextrose 5% in water, n=84) every 6 hours for 96 hours. The other half of the participants were given a placebo (dextrose 5% in water) for the same allotted period. The Sequential Organ Failure Assessment score (0-20 scale with a higher score indicative of more severe organ damage) was recorded, as well as measurements of inflammatory markers (c-reactive protein) and vascular injury (thrombomodulin levels) [58]. Surprisingly, the study concluded that there were no significant differences between the vitamin C and placebo groups in mean modified Sequential Organ Failure Assessment score from baseline to 96 hours (9.8 to 6.8 in the vitamin C group and 10.3 to 6.8 in the placebo group; *P*=.86) or in C-reactive protein levels (54.1 vs. 46.1 μg/mL; *P*=.33) and thrombomodulin levels (14.5 vs. 13.8 ng/mL; *P*=.70) at 168 hours [58]. There was however, a statistically significant difference in all-cause mortality in patients receiving IV vitamin C treatment (29.8% (25/84)) compared to the placebo group (46.3% (38/82)). Researchers proposed that this discrepancy could possibly be due to vitamin C ability to correct the underlying cause of sepsis within patients and therefore, are not reflected in the biomarker analysis; however, further research is warranted to investigate these findings.

Most recently, Fujii et al. (2020) published a multicenter, open-labeled, randomized clinical trial to investigate the efficacy of the triple therapy of vitamin C, thiamine, and hydrocortisone in increasing the time alive free of vasopressor in the patient suffering from septic shock compared to hydrocortisone treatment alone [59]. A total of 216 patients were enrolled for the trial from Australia, New Zealand, and Brazil fulfilling the sepsis-3 definition of septic shock. The patients were randomized into two groups, the study group (n=109) receiving a triple therapy consisting of intravenous vitamin C (1.5 g every 6 hours), hydrocortisone (50 mg every 6 hours), and thiamine (200 mg/12 hours), and the control group (n=107) receiving intravenous hydrocortisone (50 mg/6 hours) until shock resolution or up to 10 days. As in the CITRIS-ALI study, this study also did not find any significant improvement. This study reported that the triple therapy treatment did not show a significant increase in time alive and free of vasopressor administration compared to the control group, with the meantime being 122.1 hours and 124.6 hours, respectively. Moreover, the secondary outcome of 90-day mortality did not show any remarkable improvement with the triple therapy vs. the hydrocortisone treatment alone (28.6% vs. 24.5%, respectively) [59]. The variable results stemming from the CITRIS-ALI and Fujii et al. (2020) clinical trials raised questions on the efficacy of IV vitamin C therapy in reducing organ failure and mortality in life-threatening conditions like sepsis and ARDS. Once again, further research is warranted in order to fully know the effectiveness of vitamin C therapy in limiting the severity and duration of symptoms seen with septic patients.

## Vitamin C Supplementation for Viral Infection

The beneficial role of vitamin C as an antioxidant and anti-inflammatory is well known [56]. This leads the scientific community to conduct several clinical trials investigating whether high doses of vitamin C (>1000 mg/day) has efficacy in treating and reducing the severity of illness seen with a variety of viral infections (Table 3). In 2014, Mikirova and his group investigated the effect of early administrations of the high intravenous dose of vitamin C in patients diagnosed with Epstein-Barr virus (EBV) to reduce the duration and viral load of the disease [60]. They reported that a high dose of intravenous vitamin C administration had a positive effect on viral antibody levels and disease duration, with an inverse correlation seen with serum vitamin C levels and viral antibody levels [60]. This same positive effect was seen in another study monitoring patients with herpes zoster viral infections [61]. Once again, a high dose of Vitamin C demonstrated an ability to reduce the viral load in patients receiving the treatment, with mean declines of pain scores and fewer symptoms being reported [61]. A double-blinded randomized control trial was performed looking at whether oral ingesting of 1000 mg vitamin C supplements daily, in young adult men would reduce the severity and duration of symptoms after contracting the common cold virus [62]. Data illustrated that those participants within the study group (with 1000 mg vitamin C supplements daily) showed a reduction in incidence of contracting the common cold virus. Also, in participants who did contract the common cold virus, a significant reduction in duration and severity of symptoms (59%) was reported compared to the placebo group [62]. Several meta-analyses have been performed contradicting this result, with all concluding that vitamin C does not affect the incidence of developing a cold (For detail, see ref [11, 63, 64]). However, the studies do agree with the conclusion reach in Johnston et al. 2014, that vitamin C administration does reduce the symptoms and duration of the contracted cold but it should also be noted that in the Johnston et al. study, vitamin C was taken orally. This significantly reduces the effectiveness of high dose vitamin C therapy due to the slow absorption of ascorbic acid through the gut epithelial cells. Administration taken by this route tightly regulates the rise in serum vitamin C concentration, unlike during intravenous administration [65].

The underlying mechanism or cell signaling how Vitamin C combat the virus infection still remains unclear, but a number of theories have been proposed. Currently the rationale behind treatment with vitamin C is two-fold, with the substance having both an antioxidant and immunomodulatory affects [66]. Most viral infections are associated with decreasing levels of vitamin C well below the normal (5-15 mg/L) [67] because of the intracellular environment undergoing substantial oxidative stress. This was illustrated by researchers investigating vitamin C levels following herpes zoster infection. They found that patients who had been infected had an average serum vitamin C level of 4.6 mg/L vs. 13.5 mg/L seen in healthy individual cohort [68].It is thought that high dose vitamin C therapy helps neutralize the pro-inflammatory response and combat the elevated levels of reactive oxygen species, thus limiting collateral tissue damage that is often seen in viral infections [69]. Also, as mentioned above, vitamin C is essential for a proper and effective innate and adaptive immune response due to high concentrations of the vitamin being seen within leukocytes, lymphocytes and neutrophils [15, 16, 66]. Furthermore, vitamin C has been noted to increase chemotaxis, enhance neutrophil phagocytic capacity and support lymphocyte proliferation. Lastly, it has been shown that vitamin C levels have immunomodulatory properties in patients with viral infections due to its ability to stimulate alpha/beta interferons while simultaneously downregulating production of pro inflammatory cytokines [66, 70] . It is clear from literature that vitamin C indirectly reduce the viral load and infection through its potent antioxidant properties and immunomodulatory affects.

**Table 4:** Ongoing clinical trials register for using Vitamin C alone or in combination with other drug for treatment of COVID-19 infections.

S.No.	Identification Number	Country	Participant	Intervention	Register with
1	ChiCTR2000029768	Wuhan, Hubei, China	60 Participants	Diammonium Glycyrrhizinate Enteric-coated Capsules (oral, 150mg, Tid), Oral Vit C tablets (.5 g) every day	Chictr.org
2	ChiCTR2000030135	Xi'an, Shaanxi, China	39 Participants	High dose Vit C	WHO.int
3	NCT04264533	Wuhan, Hubei, China	140 participants	IV 12g Vit C every 12 hours	Clinicaltrail.gov
4	NCT04323514	Palermo, Italy	500 participants	IV 10g Vit C plus conventional therapy	Clinicaltrial.gov
5	NCT03680274	Sherbrooke, Quebec, Canada	800 participants	IV 50 mg/kg Vit C every 6 hours for 96 hours	Clinicaltrial.gov
6	NCT04326725	Istanbul, Turkey	80 participants	Hydroxychloroquine 200mg plus vitamin C and zinc every day	Clinicaltrial.gov
7	IRCT20190917044805N2	Tehran, Iran	60 participants	IV 12g Vit C in .5% dextrose (total volume 200ml)	IRCT.ir
8	IRCT20200324046850N5	Abadan, Khuzestan Province, Iran	40 participants	Hydroxychloroquine 200 mg plus oral 500 mg Vit C every 12 hours for 5 days	IRCT.ir
9	NCT04347889	N/A	1212 participants	Hydroxychloroquine 800 mg followed by once weekly oral hydroxychloroquine 400 mg for 3 months vs. Oral Vitamin C 1,000 mg daily	Clinicaltrial.gov
10	ChiCTR2000032400	Huangpu, Shanghai, China	120 participants	IV 100mg/kg Vit C every day	WHO.int
11	NCT04344184	Richmond, Virginia, United States	200 participants	IV 100 mg/kg Vit C every 8 hours	Clinicaltrial.gov
12	NCT04357782	Richmond, Virginia, United States	20 participants	IV 50mg/kg Vit C every 6 hours for 4 days	Clinicaltrial.gov
13	NCT04370288	Mashhad, Razavi Khorasan, Iran	20 participants	Treatment with mixture of methylene blue, Vit C, N-acetyl cysteine	Clinicaltrial.gov
14	ChiCTR2000032717	Xi'an, Shaanxi, China	60 participants	High dose vitamin C plus Chinese medicine for treatment of COVID-19	CHICTR.org.in
15	ChiCTR2000032716	Shanghai, Shanghai, China	12 participants	High dose IV vitamin C treatment upon diagnosis of severe COVID-19	CHICTR.org.in
16	NCT04363216	Philadelphia, Pennsylvania, United States	66 participants	Escalating dose of oral Vit C (0.3g/kg, 0.6g/kg, 0.9g/kg) every 6 hours	Clinicaltrial.gov
17	ACTRN12620000557932	Australia, United States, Germany	200 participants	Trial arms: 1)Hydroxychloroquine plus zinc plus Vit D3/B12 plus azithromycin plus IV Vitamin C 2) Hydroxychloroquine plus zinc plus Vit D3/B12 plus azithromycin	WHO.ir
18	IRCT20200411047025N1	Tehran, Iran	110 participants	IV 1.5g Vit C 4 times a day plus hydroxychloroquine 400 mg	IRCT.ir
19	IRCT20140305016852N4	Sabzevar, Razavi Khorasan, Iran	30 participants	Treatments of 500mg Vit C daily for a week	IRCT.ir
20	IRCT20200418047121N1	Kermanshah, Kermanshah, Iran	40 participants	250mg Azithromycin once daily, 100 mg of doxycycline twice daily, 1.5g Vit C every 6 hours, and 500mg metformin	IRCT.ir
21	TCTR20200404004	Bangkok, Thailand	400 participants	Trial arms: 1) Chloroquine 10 mg base/kg once a day 2) Vitamin C 1000 mg once a day	Clinicaltrials.in.th
22	NCT04334512	Ventura, California, United States	600 participants	Quintuple therapy consisting of hydroxychloroquine, azithromycin, zinc, vit C and D for 10 days	Clinicaltrials.gov
23	NCT04335084	Ventura, California, United States	600 participants	hydroxychloroquine, Vitamin C, Vitamin D, and Zinc can prevent symptoms of COVID-19	Clinicaltrials.gov
24	NCT04342728	Weston, Florida, United States and Cleveland, Ohio, United States	520 participants	Trial arms: 1)Vit C 8000mg in 2-3 doses 2)Zinc Gluconate 50mg daily 3)Vit C 8000mg plus Zinc gluconate 50mg daily 4)Standard of care	Clinicaltrials.gov
25	NCT04328961	Various cities throughout the united states	2000 participants	Trial arms: 1) Oral 500mg Vit C daily for 3 days then 250mg for 11 days 2) 400mg Hydroxychloroquine for 3 days then 200mg for 11 days	Clinicaltrials.gov
26	ChiCTR2000033050	Shanghai, China	110 participants	High dose IV Vitamin C to patients with COVID-19	CHICTR.org.in
27	NCT04395768	Hawthorn, Victoria, Australia	200 participants	IV vitamin C, Hydroxycholorquine, azithromycin, Zinc citrate, Vitamin D3, Vitamin B12 for treating COVID-19	Clinicaltrials.gov
28	NCT04334967	Portland, Oregon, United States	13 participants	Trial arms: 1) 800mg hydroxychloroquine on day 1, 400mg on days 2-5 2) 2000mg of Vit C on day 1, 1000mg on days 2-5	Clinicaltrials.gov
29	NCT04354428	Various cities throughout the United States	630 participants	Trial arms: 1) Vit C plus folic acid 2) Hydroxychloroquine plus Folic Acid 3) Hydroxychloroquine and Azithromycin	Clinicaltrials.gov
30	NCT04401150	Sherbrooke, Quebec, Canada	800 participants	IV 50 mg/kg Vit C every 6 hours	Clinicaltrial.gov

## COVID-19 and Vitamin C

With an exponential increase in COVID-19 infection rate and mortality in an ongoing global pandemic, researchers, clinicians, and government agencies are focusing on repurposing drugs with known safety profiles [3]. Previously known beneficial outcomes following high doses of vitamin C therapy in clinical studies have made this vitamin a frontline candidate for possible COVID-19 treatment. Also, there are very limited side effects and patients have high tolerability to ascorbic acid high doses[65]. Currently, there are approximately 30 ongoing clinical trials registered using Vitamin C alone or in combination with other drugs looking at the efficacy behind treating COVID-19 infections on Clinicaltrial.gov and International Clinical Trials Registry Platform (World Health Organization) (See Table.4). However, as of the date of writing, there are no published, peer-reviewed manuscripts or data sets looking at the effectiveness of either high dose IV or oral vitamin C in treating and limiting the symptoms of COVID-19 positive patients.

### Conclusion

Based on the literature mentioned above, high dose intravenous vitamin C therapy has been shown to have a range of effectiveness from moderate to high in preventing and limiting the duration of viral infections, with the most beneficial effect coming in those with reduced ascorbic acids levels. The vitamin C treatment is known for its beneficial role in preventing/ neutralizing inflammatory response, reducing oxidative stress, and stimulating interferons and other antiviral cytokines. Vitamin C is drug of choice in this critical time because of its known high dose tolerability and little or no side effects. It is possible that Vitamin C might help in a certain population of COVID-19 infected patients. The previous moderate success of vitamin C supplementation in human clinical studies may be due to several factors depending on the subject's age, race, levels of vitamin C transporter expression, and polymorphism in the vitamin C transporter, etc. Future clinical studies should be designed and conducted with all these factors taken into consideration, specifically vitamin C transporter expression and polymorphism. We recommend that the factors mentioned above should be considered at the start of clinical trials and during the analysis of the outcome of clinical findings. It will be interesting to see if vitamin C can help specifically in treating COVID-19 infected patients who are older, have underlying conditions or belong to African American populations. Furthermore, there is an urgent need to investigate the direct relationship between serum/plasma vitamin C levels in COVID-19 infection rate and severity.
